# A Novel Albumin-Related Nutrition Biomarker Predicts Breast Cancer Prognosis in Neoadjuvant Chemotherapy: A Two-Center Cohort Study

**DOI:** 10.3390/nu15194292

**Published:** 2023-10-08

**Authors:** Meng-Di Wang, Fang-Fang Duan, Xin Hua, Lu Cao, Wen Xia, Jia-Yi Chen

**Affiliations:** 1 Department of Radiation Oncology, Ruijin Hospital, Shanghai Jiaotong University School of Medicine, Shanghai 200025, China; wmd01f28@rjh.com (M.-D.W.); hx12914@rjh.com.cn (X.H.);; 2 The State Key Laboratory of Oncology in South China, Collaborative Innovation Center for Cancer Medicine, Sun Yat-sen University Cancer Center, Guangzhou 510060, China

**Keywords:** breast cancer, neoadjuvant chemotherapy, inflammatory–nutritional biomarker, prognosis, nomogram

## Abstract

Background: Recently, there has been a growing focus on the prognostic significance of nutrition-related biomarkers. We attempted to explore the association between a novel albumin-related nutrition marker called “lymphocyte × albumin (LA)” and disease-free survival (DFS) in breast cancer patients undergoing neoadjuvant chemotherapy (NAC). Methods: In total, 711 non-metastatic breast cancer patients who underwent NAC at two medical centers were retrospectively analyzed. We performed least absolute shrinkage and selection operator (LASSO) Cox regression analysis as well as multivariate Cox regression analyses to identify the variables associated with DFS and to establish a predictive nomogram. Results: The nomogram incorporated four variables based on the multivariate analysis of DFS in the training cohort: LA, ypN stage, ypT stage, and hormone receptor status. In comparison with the traditional TNM staging system, the nomogram demonstrated superior discrimination, calibration ability, and clinical usefulness in both the training set and internal and external validation sets. Furthermore, patients stratified into different risk groups resulted in significant differences in DFS. Conclusions: LA is an independent prognostic biomarker, and LA-based prognostic nomogram offers a more precise assessment of DFS for breast cancer patients treated with NAC, potentially serving as a valuable tool for personalized prognostic predictions.

## 1. Introduction

Globally, breast cancer is the most frequently diagnosed malignancy and the leading cause of cancer death among women [1]. Approximately 2.3 million new cases of breast cancer are diagnosed each year, and 685,000 people die from it [1]. In the treatment of breast cancer, neoadjuvant chemotherapy (NAC) has brought new hope with breakthroughs in effective treatment. Nowadays, the clinical advantages of NAC are not only to achieve tumor down-staging and breast conservation surgery, but also to provide an opportunity for in vivo drug-sensitivity testing [2]. While researchers have validated the efficacy of NAC, the presence of high intertumoral heterogeneity leads to diverse outcomes for individual breast cancer patients [3]. Recurrence and metastasis may still occur in breast cancer patients who have undergone curative surgery and received neoadjuvant/adjuvant therapy [4]. Therefore, the discovery of reliable prognostic biomarkers holds paramount significance in developing risk stratification methods and planning appropriate treatment in advance for patients, ultimately leading to maximal improvements in survival outcomes. 

In newly diagnosed breast cancer patients, traditional clinicopathological factors such as lymph node metastases, tumor size, and grade have been utilized to provide individual prognostic information. However, it is widely acknowledged that relying solely on these factors for personalized therapeutic guidance is insufficient. Combining multigene gene tests, biomarkers such as estrogen receptor (ER), progesterone receptor (PR), human epidermal growth factor receptor-2 (HER2), and traditional pathological clinical prognostic factors can establish better prognostic models [5,6]. Although several gene expression assays, including Oncotype DX, MammaPrint, EndoPredict, Prosigna/PAM50, and Breast Cancer Index (BCI), have been validated to define prognosis more accurately and recommended to clinical practice in recent decades, the costs of these tests are quite expensive in many countries [7,8]. Therefore, the exploration of simple, affordable, and accurate prognostic biomarker assays has become a research hotspot.

An increasing amount of evidence highlights the interconnection of inflammation, nutrition, and breast cancer, impacting the processes of initiation, progression, and metastasis [9,10]. In recent years, many inflammatory and nutritional prognostic indices, including the lymphocyte-to-monocyte ratio (LMR) [11,12], prognostic nutrition index (PNI) [13,14], nutritional risk index (NRI) [15], and pan-immune-inflammation value (PIV) [16,17], have emerged as potential prognostic factors for breast cancer. These prognostic indices possess several advantages, including cost-effectiveness, quick accessibility, ease of calculation, reproducibility, and widespread availability. They can be readily obtained from pretreatment blood tests, which are routinely conducted for nearly all cancer patients. The novel albumin-related nutrition biomarker “LA”, which was defined as lymphocyte × albumin, was firstly introduced in rectal cancer patients [18]. From a theoretical perspective, LA shares similarities with PNI, yet LA offers the advantage of easier calculation and simpler utilization for patient stratification [19]. However, there remains insufficient evidence to determine whether the above-mentioned indices can be applied as a whole to breast cancer patients receiving NAC. Accordingly, we specifically designed the present study to address these particular concerns.

## 2. Materials and Methods

### 2.1. Patients

**Figure 1:**
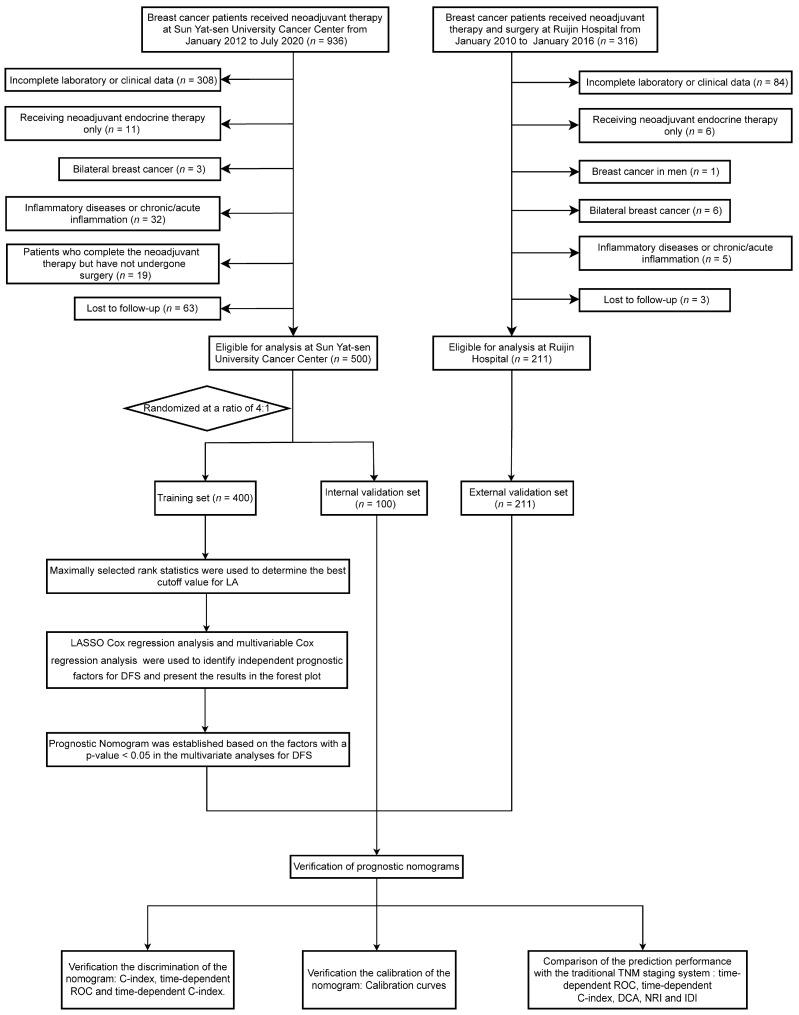
Study design flowchart. Abbreviations: LASSO, least absolute shrinkage and selection operator; DFS, disease-free survival; ROC, Receiver Operating Characteristic Curve; DCA, decision curve analysis; NRI, net reclassification index; IDI, integrated discrimination improvement; C-index, Harrell’s concordance index.

In our study, we retrospectively screened 1252 patients with breast cancer who received neoadjuvant therapy at two hospitals: 936 patients at Sun Yat-sen University Cancer Center (SYSUCC) from January 2012 to July 2020 and 316 patients at Ruijin Hospital from January 2010 to January 2016. The following criteria were used for inclusion: (1) Patients with non-metastatic breast cancer aged ≥18 years who received NAC. (2) Before NAC, all patients underwent ultrasound-guided diagnostic core needle biopsy of the primary tumor. Additionally, for lymph nodes suspected to be positive on ultrasound examination, the nodal status was determined by fine needle aspiration. (3) All patients underwent rigorous clinical imaging examinations (e.g., ultrasound, CT, MRI, etc.) before NAC and were ultimately diagnosed with stage I-III breast cancer. (4) Patients’ laboratory data before NAC were available. (5) Follow-up and clinical pathology information for the patients were complete. The following criteria were used for exclusion: (1) Patients with bilateral or inflammatory breast cancer. (2) Male patients. (3) Patients who were treated solely with neoadjuvant endocrine therapy. (4) Patients who completed the NAC but had not undergone surgery. (5) Patients with inflammatory diseases or chronic/acute inflammation, including autoimmune diseases. (6) Patients with a history of malignancies at other sites. Finally, a total of 711 patients were analyzed in the present study. Specifically, 500 patients from Sun Yat-sen University Cancer Center were allocated into training and internal validation cohorts randomly, with a ratio of 4:1. Concurrently, an additional 211 patients from Ruijin Hospital were assigned to the external validation set. The patient inclusion process and the study design are shown in Figure 1. The Research Ethics Committee of Sun Yat-sen University Cancer Center and Ruijin Hospital approved the present retrospective study, and confidentiality was maintained for the data of all patients.

### 2.2. Data Collection and Classification

We collected the following variables from the electronic medical records of all participants: age; menopausal status; pre-treatment histological types; pre-treatment histological grade; T stage before NAC (cT Stage); N stage before NAC (cN Stage); T stage after surgery (ypT Stage); N stage after surgery (ypN Stage); TNM stage after surgery (ypTNM Stage); pathologic complete response (pCR); HR status; HER2 status; Ki-67; lymphovascular invasion; type of primary surgery; and NAC regimens. Immunohistochemical analysis (IHC) was applied to determine the ER and PR statuses, with a positive classification for hormone receptors (HRs) being assigned when the cells staining ER- or PR-positive exceeds 1%. If the gene amplification ratio by fluorescence in situ hybridization was greater than 2.2 or the expression level intensity on IHC was 3+, the HER2 status was considered to be positive. The cutoff value for Ki67 level was based on 14% of tumor cells showing nuclear staining [20]. The absence of invasive carcinoma in both regional lymph nodes and breast tissue could be recognized as pCR, with the allowance of residual ductal carcinoma in situ (DCIS) within the breast tissue [21]. pCR was assessed by reviewing the final pathological reports of all patients. According to the American Joint Committee on Cancer—Tumor, Node, and Metastases (AJCCTNM) staging system 8th edition, at the end of the follow-up, all patients were restaged.

The body mass index (BMI) and primary laboratory data were collected within two weeks before NAC (at first diagnosis before any treatment). LA was computed using the formula provided below: LA = lymphocyte count × serum albumin concentration (g/L). Weight (kg) divided by the square of height (m) was the formula used to calculate BMI. The LA and BMI were computed using Microsoft Excel. The optimal cutoff value for LA was 49.06 in the training cohort by using maximally selected rank statistics [22] (Figure S1).

### 2.3. Follow-Up and Endpoints

Telephone follow-up or outpatient electronic records were used to monitor patients’ medical conditions regularly. In cases where patients had passed away, the cause and date of death were also recorded during the follow-up process. For the first two years, patients had evaluations every three months. From year 2 to year 5, they had evaluations every six months, and subsequently once a year. Routine breast and abdominal ultrasonography or computed tomography, monitoring of menstrual status, and hematological and laboratory examinations were all included in the assessments. Additionally, annual bone scans and X-rays were conducted.

DFS was the study’s primary endpoint. It was calculated by measuring the time in months that had passed between the date of curative surgery and the first event, which could be either disease recurrence or death, whichever came first. Overall survival (OS), the secondary endpoint, was calculated by measuring the number of months between the date of curative surgery and the occurrence of death due to any cause. For patients who did not experience any recurrence or mortality events during the follow-up period, the date of their last follow-up was the study’s endpoint.

### 2.4. Statistical Analysis

Due to the absence of data for constructing prognostic models, prior sample size calculations were not conducted. Nevertheless, there were 711 participants enrolled in the study, and the total number of events was 205. In the multivariate models, this resulted in an excess rate of 10 events per variable, indicating an adequate level of statistical power for the evaluation [23]. Laboratory data from SYSUCC and Ruijin Hospital were normalized and batched using the limma R package. For continuous variables, the median was used for presentation. Meanwhile, categorical variables were displayed using both frequencies and percentages. Either Pearson’s chi-square test or Fisher’s exact test were used to compare these variables. Statistical significance was defined as a two-tailed *p*-value < 0.05. The optimal cutoff value of LA was determined using the “maxstat” package in the R software version 4.2.2, employing maximally selected rank statistics with survival status as the endpoint. The Kaplan–Meier method was used to construct survival curves, and the log-rank test was used to compared them. The proportional hazards assumption test was conducted based on the Schoenfeld residuals. Univariate and multivariate analyses were performed employing the Cox proportional hazards model to derive hazard ratios (HRs) and 95% confidence intervals (CIs). Specifically, in the univariate analysis, variables with *p*-values below 0.05 were included in the multivariate analysis to identify independent risk factors. Given the potential for multicollinearity in the training set, we employed the “glmnet” package in R software to perform least absolute shrinkage and selection operator (LASSO) Cox regression analysis in order to identify the most significant factors. In the LASSO Cox model, we applied an L1 penalty to precisely reduce certain regression coefficients to 0. Additionally, we conducted a 10-fold cross-validation to determine the optimal value of log(λ), which is a tuning parameter used to control the degree of shrinkage. To identify independent prognostic factors for DFS, all factors that have non-zero coefficients in the LASSO analysis will be integrated into the multivariable Cox regression analysis. 

Only the factors that demonstrated statistical significance with a *p*-value < 0.05 in the multivariate analyses were chosen to construct the prognostic model in the training set. In R software, this model was visually depicted as a nomogram through the utilization of the “rms” package. The model’s discrimination ability was assessed through Harrell’s concordance index (C-index), time-dependent C-index curve, and time-dependent receiver operating characteristic (ROC) curve. The calibration performance was assessed using calibration curves, while the internal validity of the prediction models was examined using the bootstrap method. Through the application of decision curve analysis (DCA), the practical value of the prognostic model was evaluated [24]. The assessment of clinical benefits and the utility of the nomogram model in comparison to the traditional AJCC-TNM staging system was performed using the net reclassification index (NRI) and integrated discrimination improvement (IDI). These measures further illustrated the superior risk prediction and usefulness of our models [25,26]. It signifies a positive enhancement if NRI and IDI are greater than 0, suggesting that the new model’s predictive capability has improved in comparison to the old model. Conversely, it signifies a negative change if NRI and IDI are less than 0, indicating a reduction in the predictive accuracy of the new model [27]. To illustrate the differences in survival time, population distribution, and research indicators between high- and low-risk groups, a risk plot was utilized for visualization [27,28]. R software (http://www.R-project.org; accessed date: 8 November 2022 version 4.2.2) and SPSS 26.0 (IBM Corporation, Armonk, NY, USA) were used to conduct the statistical analyses.

## 3. Results

### 3.1. Patient Characteristics

In this study, 500 breast cancer patients who met the specified inclusion and exclusion criteria and were scheduled to receive NAC prior to surgery at SYSUCC between January 2012 and July 2020 were enrolled. In a ratio of 4:1, the entire set of cases was randomly split into two groups: the training cohort, consisting of 400 cases, and the internal validation cohorts, consisting of 100 cases. The baseline clinicopathological characteristics are presented in Table 1. Although some significant differences existed in ypT stage between the groups, they were generally comparable. Upon diagnosis, the median age for all patients was 48.0 years, and over two-thirds of them were premenopausal women. The groups did not show any significant variations in terms of BMI. Most of the enrolled patients were pathologically diagnosed with invasive ductal carcinoma (IDC), HR-positive (65.80%), and lymphovascular invasion (61.00%), and 420 (84.00%) patients had a Ki-67 index value ≥ 14. There were 216 cases (43.20%) of HER-2-positive patients among the total population. More than 80% of the patients received NAC regimens containing both anthracycline and taxane. According to the AJCC-TNM classification system eighth edition, the patients’ clinical staging was predominantly at stage II–III. In the overall population, as for the postoperative pathological staging, ypT1–T2 stage (65.60%) and ypN0–N1 stage (67.80%) were the most common. Eighty-six cases (17.20%) achieved pCR. Based on the optimal LA cut-off value of 49.06, 51 (12.80%) in the training set and 13 (13.00%) patients in the internal validation set were assigned to the low-LA groups (<49.06). Correspondingly, 349 patients (86.20%) and 87 patients (87.00%) were classified into the high-LA group (≥49.06). Table S1 shows that there was no significant correlation between LA and different clinicopathological factors in the training cohort.

### 3.2. Prognostic Value of LA 

**Figure 2:**
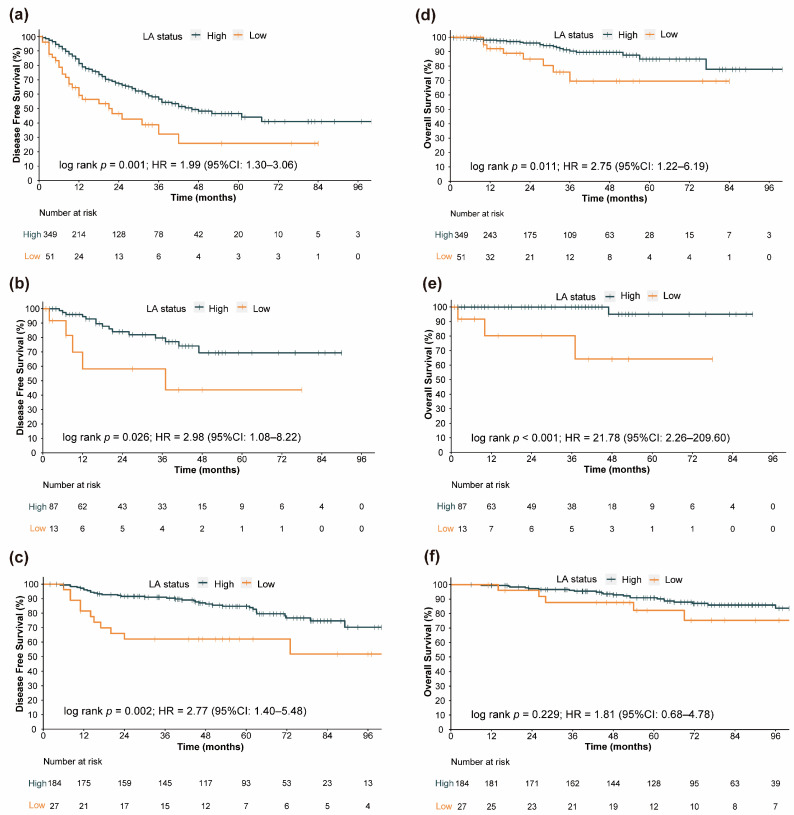
Kaplan–Meier curves of various survival endpoints between the high-LA group and low-LA group. For disease-free survival in different LA level groups: (**a**) Training cohort survival curves. (**b**) Internal validation cohort survival curves. (**c**) External validation cohort survival curves. For overall survival in different LA level groups: (**d**) Training cohort survival curves. (**e**) Internal validation cohort survival curves. (**f**) External validation cohort survival curves.

The median follow-up in the entire cohort was 41 months (95% CI: 38.80–45.00 months). In the training cohort, 140 recurrence or death events were observed. The internal validation cohort experienced 20 such events, and the external validation cohort had 45 of them. In our study, “Number at risk” represents the remaining number of patients exposed to the outcome risk at the corresponding time point. Generally, at the 1-year and 2-year time points, the low-LA group exhibited a higher rate of recurrence or death events. In Figure 2, significantly longer DFS in the high-LA group than in the low-LA group (Figure 2a, hazard ratio [HR] = 1.99, 95% CI: 1.30–3.06, log-rank test *p* = 0.001; Figure 2b, HR = 2.98, 95% CI: 1.08–8.22, log-rank test *p* = 0.026; Figure 2c, HR = 2.77,95% CI: 1.40–5.48, log-rank test *p* = 0.002) was shown in the Kaplan–Meier curves, whether in the training cohort, internal validation cohort, or external validation cohort. 

Additionally, we attempted to explore the association between LA and OS. As shown in Figure 2d,e, in both the training cohort and the internal validation cohort, the OS of the low-LA group was significantly worse than that of the high-LA group (Figure 2d, HR = 2.75, 95% CI: 1.22–6.19, log-rank test *p* = 0.011; Figure 2e, HR = 21.78, 95% CI: 2.26–209.60, log-rank test *p* < 0.001). However, possibly influenced by sample size and follow-up duration, the survival curves exhibited a similar trend, but the *p*-value failed to reach statistical significance in the external validation cohort (Figure 2f, HR = 1.81, 95% CI: 0.68–4.78, log-rank test *p* = 0.229).

### 3.3. Development of the Prognostic Model

**Figure 3:**
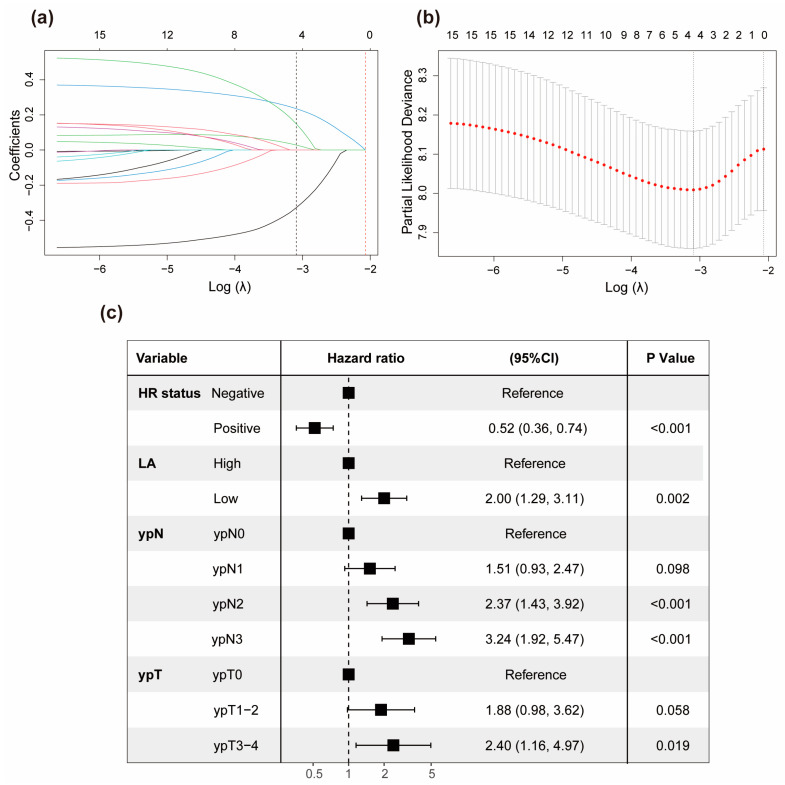
Significant factors determination using LASSO Cox and multivariate Cox regression model. (**a**) A coefficient profile plot of 15 factors associated with DFS in the training set. The bottom horizontal axis represented the log lambda (λ) values of the independent variable, the top horizontal axis indicated the number of variables with non-zero coefficients, and the vertical axis represented the coefficients of the independent variable. Different-colored curves represented different variables, with each curve depicting the trajectory of the coefficient variation for each independent variable. The black dotted vertical line (left), positioned at the λ value where the bias was minimized, indicated the optimal model fitting point with four non-zero coefficients. The red dotted vertical line (right) was located at one standard error to the right of the minimum lambda value. (**b**) Four non-zero coefficient variables selected through ten-fold cross-validation via optimal lambda. Dotted vertical lines were placed at the optimal log(λ) values determined by both the minimum criteria and one standard error. (**c**) Multivariate Cox regression analysis on the four variables selected through Lasso Cox regression presented in a forest plot. Abbreviations: HR, hormone receptors; CI, confidence interval.

**Figure 4:**
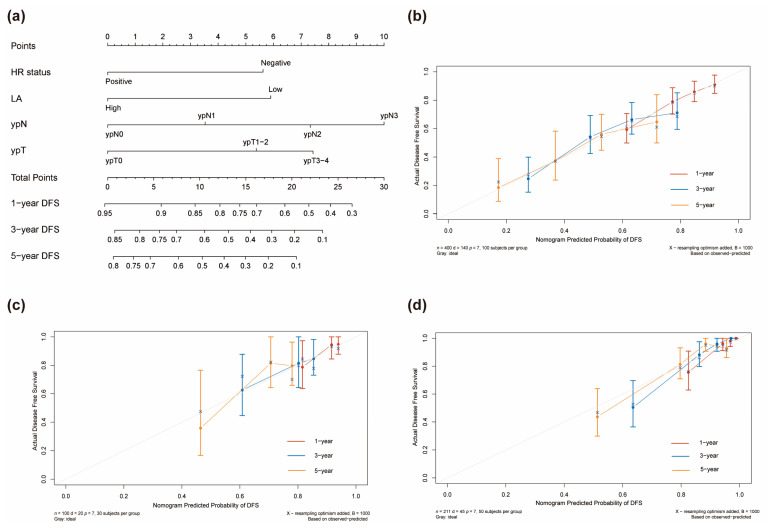
Nomograms for prognostic model development and calibration. (**a**) A nomogram to predict the 1-, 3-, and 5-year DFS of breast cancer patients with NAC. Calibration plot in the training (**b**), internal validation, (**c**) and external validation (**d**) cohorts.

Table S2 displays the univariable analysis of DFS via Cox regression analysis in the training cohort. cT Stage, ypN Stage, ypT Stage, pCR, LA, and HR status are the factors of statistical significance (*p* < 0.05). We used the LASSO Cox regression model to address the problem of multicollinearity upon regression in the training cohort (Figure 3a,b). Subsequently, at the optimal lambda value with minimal bias, we identified four indicators associated with DFS, each having non-zero coefficients. These indicators included ypT stage, ypN stage, HR status, and LA, all of which were incorporated into the multivariate Cox regression model. In the multivariable modeling, the proportional hazards diagnostic tests confirmed that the assumption of proportional hazards was met (Figure S2). Finally, multivariable Cox regression analysis demonstrated that LA had the capability to predict DFS independently (HR = 2.00, 95% CI: 1.29–3.11, *p* = 0.002). Furthermore, negative hormone receptor status, larger ypT stage, and elevated ypN stage were all found to be independent predictors of adverse DFS for breast cancer patients who underwent NAC. Furthermore, we visually depicted the aforementioned multivariate Cox regression outcomes using a forest plot (Figure 3c). Afterwards, a novel prognostic model based on those aforementioned four independent prognostic factors was established and visually represented as a nomogram for predicting the 1-, 3-, and 5-year DFS of breast cancer patients with NAC (Figure 4a). A corresponding score was assigned to each prognostic factor, and by summing up the scores associated with all the prognostic factors, the total score for each individual was determined. The total score was then located on the survival rate scale to predict the 1-, 3-, and 5-year DFS rate of the patients before the implementation of subsequent adjuvant therapy. It was evident that higher total scores in patients were associated with a decreased probability of individual DFS.

### 3.4. Assessment of Predictive Performance of the Prognostic Model

**Figure 5:**
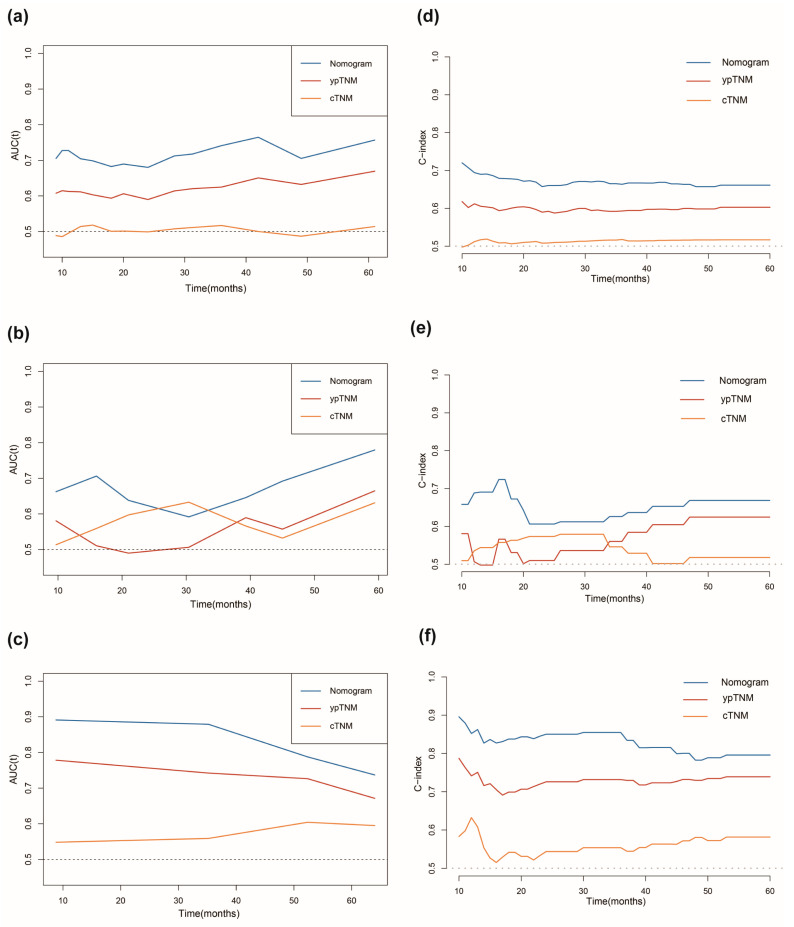
Comparison of the predictive capabilities between the current prognostic model and traditional TNM staging. Time−dependent ROC curves comparison in the training cohort (**a**), internal validation cohort (**b**), and external validation cohort (**c**). Time−dependent C−index curves comparison in the training cohort (**d**), internal validation cohort, (**e**) and external validation cohort (**f**).

**Figure 6:**
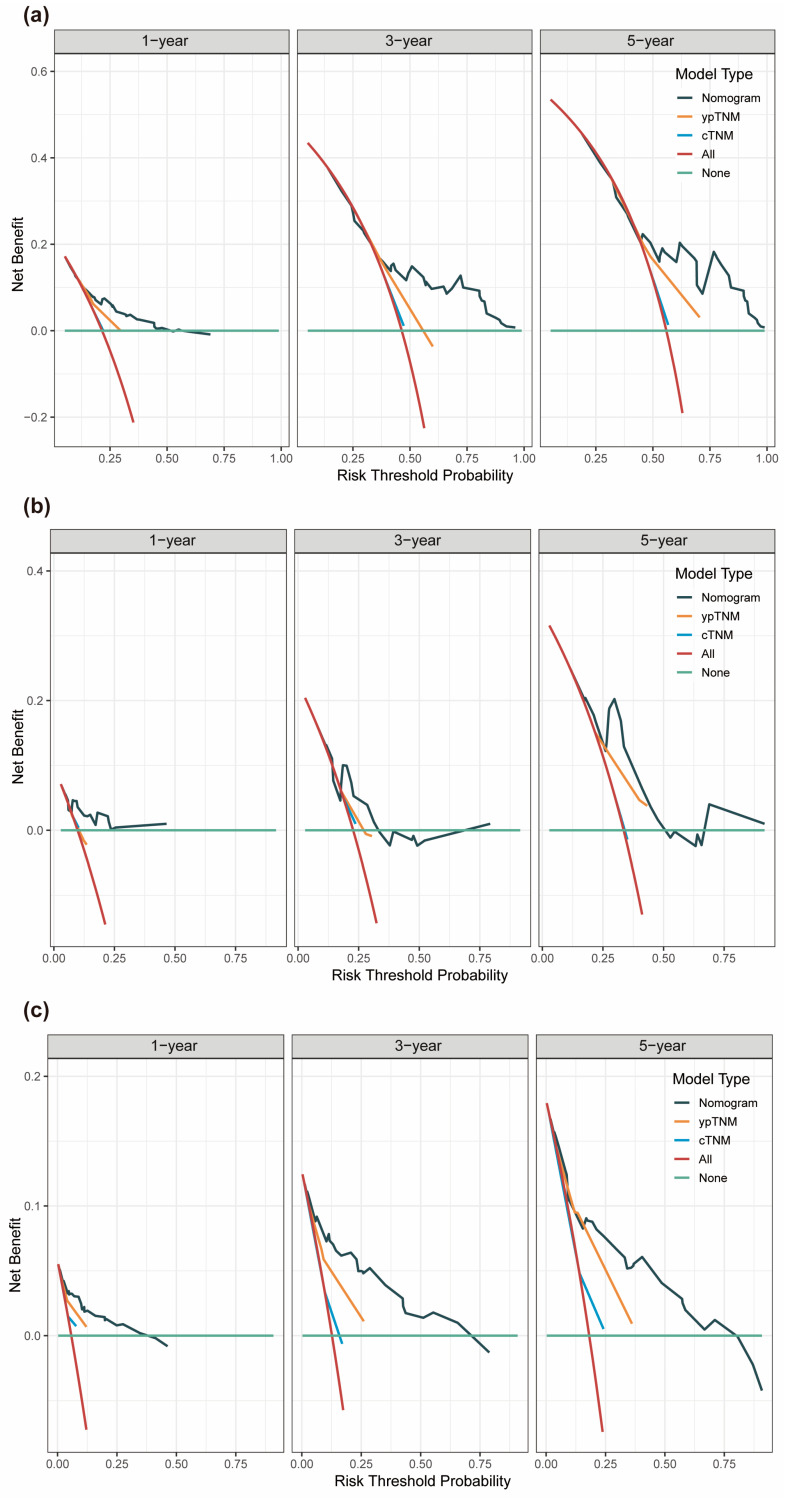
Time−dependent decision curve analysis (DCA) curves were plotted based on 1-, 3-, 5-year DFS benefit. DCA for the nomogram, cTNM and ypTNM models in the training set (**a**), internal validation set (**b**), and external validation set (**c**).

Strong discriminatory performance was demonstrated by the generated prognostic model with a *C*-index of 0.69 (95% CI: 0.64–0.74) for the training cohort, 0.66 (95% CI: 0.51–0.81) for the internal validation cohort, and 0.80 (95% CI: 0.74–0.87) for the external validation cohort. As shown in Figure 4b–d, the calibration curves of the 1-, 3-, and 5-year DFS probability nomograms exhibited strong agreement between the predicted and observed survival probabilities across the training, internal, and external validation sets. To assess the prognostic accuracy of this customized DFS prognostic model, time-dependent ROC analysis was conducted. Across the training, internal, and external validation cohorts, our predictive nomogram for individualized DFS, as depicted in Figure 5a–c, showed superior performance compared to the traditional TNM stage. In addition, we also plotted time-dependent *C*-index curves, which further confirmed the above results (Figure 5d–f). Furthermore, we conducted an accuracy comparison between the nomogram and the TNM stage using IDI and NRI in the training, internal, and external validation cohorts (Table 2). While in different cohorts, both NRI and IDI were greater than 0, indicating a positive improvement, suggesting that the nomogram demonstrated superior predictive accuracy in prognosis when compared to the conventional staging system. Through DCA, a notable improvement in the net benefit of the proposed model when compared to the AJCC tumor staging system was observed in the training, internal, and external validation cohorts (Figure 6). Compared to the AJCC staging system, these results highlight that the new nomogram model holds greater clinical utility in accurately predicting individual survival outcomes.

### 3.5. Performance of the Nomogram in Risk Stratification of Patients

**Figure 7:**
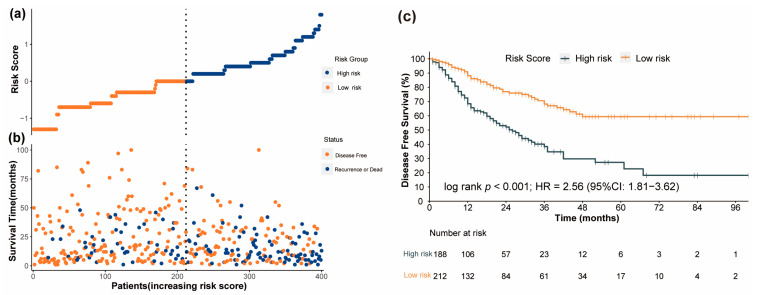
The performance of risk stratification using the prognostic nomogram model in the training cohort (“Number at risk” represents the remaining number of individuals exposed to the outcome risk at the corresponding time point). (**a**) The distribution and median value of the risk scores. (**b**) The distribution of DFS, DFS status, and risk score. (**c**) The Kaplan–Meier curves for the DFS of patients who were divided into high−risk and low−risk groups.

In the training cohort, the prognostic nomogram model was utilized to compute a score for each patient, and the patients were categorized into either a low-risk or a high-risk group based on their median score (Figure 7a). In Figure 7b, it is indicated that individuals with high-risk scores have a greater likelihood of experiencing disease progression or death compared to those with low-risk scores. Similarly, breast cancer patients in the high-risk group exhibited markedly inferior DFS compared to those in the low-risk group, as illustrated by the Kaplan–Meier survival curve in Figure 7c (*p* < 0.001). Then, the internal and external validation cohorts were also separately split into a high-risk group and a low-risk group based on the median score of each cohort. In both internal and external validation cohorts, it was further confirmed that patients belonging to the high-risk group experienced worse DFS outcomes (Figures S3 and S4).

## 4. Discussion

This study represents, to the best of our knowledge, the first investigation into the prognostic significance of a novel nutritional biomarker, LA, in breast cancer patients undergoing NAC. In breast cancer patients who underwent NAC and mastectomy, multivariable Cox regression analysis demonstrated that pre-NAC LA had the ability to predict DFS, leading to the development of a novel and user-friendly nomogram model for predicting DFS. This model incorporates LA and three other significant clinicopathological variables (ypN stage, ypT stage, hormone receptor status). In comparison with the TNM staging system, the nomogram demonstrated superior discrimination, calibration ability, and clinical usefulness in all the datasets, including the training set as well as the internal and external validation sets. The validation in both internal and external patient cohorts supported the generalizability of the nomogram. Given the limited accuracy of prognosis prediction in breast cancer patients undergoing NAC solely based on traditional TNM staging, the LA-based nomogram model emerges as a reliable, cost-effective, readily accessible, non-invasive, and easily applicable alternative. It holds the potential to offer personalized prognostic recommendations for this heterogeneous patient population.

The importance of inflammation–nutrition-related biomarkers in predicting the prognosis of breast cancer patients undergoing neoadjuvant therapy has become a research hotspot in recent years [17,29,30,31]. Chen et al. found that the systemic immune-inflammation index serves as a crucial prognostic factor for breast cancer patients, offering a reliable means to predict the survival in those receiving NAC. Patients with a higher SII value experienced shorter OS and DFS [30]. In 2021, Ahmet Bilgehan Şahin et al. introduced a biomarker for systemic immune-inflammation score known as the pan-immune inflammation value (PIV). This marker, which takes the neutrophil, platelet, monocyte, and lymphocyte into account, has been demonstrated to be a better predictor of response to chemotherapy and survival of breast cancer patients who have undergone NAC. Patients in the lower PIV group exhibited remarkably improved DFS and OS [17]. Zhu et al. pointed out that the systemic inflammation response index (SIRI) could predict survival in breast cancer patients who underwent NAC independently. Patients with higher SIRI scores experienced significantly shorter DFS and OS [31]. Another previous study demonstrated that the prognostic nutritional index (PNI) served as a valuable prognostic indicator for breast cancer patients undergoing NAC. Higher PNI values indicated longer DFS and OS in breast cancer patients [29]. In rectal cancer patients with stage II/III disease who underwent radical resection, Yamamoto et al. identified a novel prognostic marker, “lymphocyte × albumin (LA),” which was significantly associated with patient survival. Lower levels of LA were indicative of poorer recurrence-free survival and OS [18]. Additionally, Yamamoto et al. also proposed that LA could potentially function as a prognostic marker for individuals diagnosed with colorectal cancer [19]. In lung cancer patients, Chen et al. confirmed lower LA levels compared to a healthy control group, and as the disease advanced, LA levels demonstrated a declining trend. Consequently, LA arises as a prospective supplementary biomarker for the diagnosis of lung cancer and may partially signify the progression of the disease [32]. Theoretically, our novel prognostic marker “LA” shares similarities with PNI; however, LA offers the advantage of being a more straightforward calculation, which can provide opportunities for further clinical practice. Compared to previous single-center studies with relatively small sample sizes, we confirmed the independent association of higher LA values with longer DFS in a cohort of 711 patients undergoing NAC for breast cancer derived from two different medical centers. In addition, we also constructed prognostic nomograms based on LA in the training group, which had a greater predictive ability for DFS than traditional TNM staging, and validated that this prognostic nomogram better predicted individual survival in the both the internal and external validation group.

Although the underlying causal effects of the association between LA and prognosis remain unclear, several hypotheses can be proposed. Lymphocytes are potent executors of the host’s anti-tumor immunity and immune surveillance functions. Higher peripheral blood lymphocyte counts indicate the endogenous anti-tumor capacity of the body, with CD8+ T lymphocytes being the primary effector cells of the anti-tumor immune response. Cytotoxic CD8+ T lymphocytes not only directly kill tumor cells through perforin and granzyme pathways or the Fas/Fas ligand (FasL) pathway but also indirectly eliminate tumor cells by secreting cytokines such as IFN-γ and TNF-α [33]. Elevated levels of lymphocytes in peripheral blood reflect the inherent anti-tumor capability of the immune system and have been linked to favorable prognoses in breast cancer [34,35,36]. Human serum albumin, primarily produced and secreted by the liver, is the most abundant protein in plasma [37]. As early as the mid-20th century, researchers discovered that tumors are capable of capturing plasma proteins and utilizing their degradation products for proliferation [38]. Serum albumin exhibits a higher turnover rate within tumor tissues, which serves as an important source of energy and nutrients that support tumor growth. In late-stage cancer patients, serum albumin synthesis is suppressed by malnutrition and inflammation. As a component of the body’s inflammatory response triggered by the tumor, the production of proinflammatory cytokines (such as IL-6) may be a contributing factor to the lower serum albumin concentration. On the other hand, the tumor necrosis factor could potentially enhance the permeability of the microvasculature, thereby facilitating the greater passage of albumin across the capillaries. As a consequence, in cancer patients, measuring the levels of serum albumin in the blood can reflect the severity of the disease, the nutritional status, the disease progression, and the prognosis [39,40]. It is widely acknowledged that serum albumin level independently plays a significant role in predicting survival outcomes in various types of cancers, such as lung, pancreatic, gastric, hepatocellular, and breast cancer [37,39,40]. These results suggested that LA might be a useful indicator of both the immunological response, as shown by the lymphocyte count, and the nutritional condition, as indicated by the serum albumin levels [18,19]. Our findings confirm that in breast cancer patients who underwent NAC, a heavy tumor burden may lead to a reduction in LA, which has been linked to a poorer prognosis.

The results of this study indicated that ypN staging, rather than cN staging, is an independent predictor for DFS. Among patients undergoing NAC, a progressively higher pathological lymph nodal involvement (ypN) correlates with a sustained escalation in the risk of DFS events. These findings are consistent with previous research results [41,42,43]. Additionally, based on the results of Harrell’s C-index, time-dependent ROC curve, and time-dependent C-index curve, ypTNM staging demonstrated better performance compared to cTNM. Therefore, post-NAC pathological staging may be more important in predicting patients’ survival risk compared to the initial clinical staging. Initial clinical staging may better reflect the initial tumor burden, while pathological staging reflects the effectiveness of NAC and the subsequent good control of local and distant diseases, indicating the concept of response-adaptive therapy [43].

However, there are some limitations worth noting in the present work. Firstly, it is a retrospective study, which may introduce potential biases in the data selection and analysis. Secondly, even though this study employed strict inclusion and exclusion criteria, it is important to recognize that serum markers can be influenced by various other factors; thus, it is essential to exercise caution when interpreting the results of our study. Despite being a two-center study, more large and well-designed studies should be performed to address these concerns. Hence, in the future, we intend to gather additional data for dynamic analysis to yield more comprehensive results. Additionally, we are in the process of planning further prospective studies to validate these findings.

## 5. Conclusions

In conclusion, the present study demonstrated that the novel nutrition–inflammation marker LA could serve as an independent prognostic indicator for the DFS time of breast cancer patients undergoing NAC. A low pre-treatment LA is associated with poorer survival compared to a high LA level. Moreover, the LA-based prognostic nomogram not only showed satisfactory discrimination and consistency but also displayed superior predictive capabilities in comparison to the conventional staging system. It holds the potential to offer personalized prognostic recommendations for breast cancer patients treated with NAC.

## Figures and Tables

**Table 1:** Patient clinical characteristics and demographics between the training and internal validation cohorts.

Variables	Total (*n* = 500)	Training Set (*n* = 400)	Internal Validation Set (*n* = 100)	*p*
**Age (years)**				
median (IQR)	48 (40, 56)	48 (40, 56)	46 (39, 55)	0.152
≤50	298 (59.60%)	236 (59.00%)	62 (62.00%)	0.665
>50	202 (40.04%)	164 (41.00%)	38 (38.00%)	
**BMI (kg/m^2^)**				
median (IQR)	23.0 (21.3, 25.0)	23.0 (21.4, 25.0)	22.8 (20.7, 24.9)	0.121
≤25	378 (75.6%)	302 (75.5%)	76 (76.0%)	1.000
>25	122 (24.4%)	98 (24.5%)	24 (24.0%)	
**Menopausal status**				
Pre-menopausal	306 (61.20%)	244 (61.00%)	62 (62.00%)	0.945
Post-menopausal	194 (38.80%)	156 (39.00%)	38 (38.00%)	
**Histological types**				
IDC	466 (93.20%)	372 (93.00%)	94 (94.00%)	0.101
ILC	14 (2.80%)	11 (2.80%)	3 (3.00%)	
Others	17 (3.40%)	16 (4.00%)	1 (1.00%)	
Missing data	3 (0.60%)	1 (0.20%)	2 (2.00%)	
**Histological grade**				
I	8 (1.60%)	5 (1.20%)	3 (3.00%)	0.217
II	231 (46.20%)	192 (48.00%)	39 (39.00%)	
III	156 (31.20%)	124 (31.00%)	32 (32.00%)	
Missing data	105 (21.00%)	79 (19.80%)	26 (26.00%)	
**cT Stage**				
T1	21 (4.20%)	19 (4.80%)	2 (2.00%)	0.176
T2	288 (57.60%)	225 (56.20%)	63 (63.00%)	
T3	111 (22.20%)	95 (23.80%)	16 (16.00%)	
T4	80 (16.00%)	61 (15.20%)	19 (19.00%)	
**cN Stage**				
N0	20 (4.00%)	14 (3.50%)	6 (6.00%)	0.186
N1	75 (15.00%)	56 (14.00%)	19 (19.00%)	
N2	290 (58.00%)	241 (60.20%)	49 (49.00%)	
N3	115 (23.00%)	89 (22.20%)	26 (26.00%)	
**ypT Stage**				
Tis/T0	98 (19.60%)	75 (18.80%)	23 (23.00%)	0.790
T1	135 (27.00%)	107 (26.80%)	28 (28.00%)	
T2	193 (38.60%)	156 (39.00%)	37 (37.00%)	
T3	45 (9.00%)	37 (9.20%)	8 (8.00%)	
T4	29 (5.80%)	25 (6.20%)	4 (4.00%)	
**ypN Stage**				
N0	215 (43.00%)	159 (39.80%)	56 (56.00%)	0.008
N1	124 (24.80%)	108 (27.00%)	16 (16.00%)	
N2	86 (17.20%)	75 (18.80%)	11 (11.00%)	
N3	75 (15.00%)	58 (14.50%)	17 (17.00%)	
**pCR**				
No	414 (82.80%)	335 (83.80%)	79 (79.00%)	0.328
Yes	86 (17.20%)	65 (16.20%)	21 (21.00%)	
**HR status**				
Negative	171 (34.20%)	135 (33.80%)	36 (36.00%)	0.759
Positive	325 (65.80%)	263 (66.30%)	62 (64.00%)	
**HER2 status**				
Negative	272 (54.40%)	220 (55.00%)	52 (52.00%)	0.161
Positive	216 (43.20%)	173 (43.20%)	43 (43.00%)	
Missing data	12 (2.40%)	7 (1.80%)	5 (5.00%)	
**Ki-67 (%)**				
median (IQR)	30 (20, 50)	30 (20, 53)	30 (20, 40)	0.274
≤14	69 (13.80%)	52 (13.00%)	17 (17.00%)	0.466
>14	420 (84.00%)	340 (85.00%)	80 (80.00%)	
Missing data	11 (2.20%)	8 (2.00%)	3 (3.00%)	
**Lymphovascular invasion**				
No	305 (61.00%)	238 (59.50%)	67 (67.00%)	0.369
Yes	187 (37.40%)	155 (38.80%)	32 (32.00%)	
Missing data	8 (1.60%)	7 (1.80%)	1 (1.00%)	
**Type of primary surgery**				
Mastectomy	450 (90.00%)	363 (90.80%)	87 (87.00%)	0.351
BCS	50 (10.00%)	37 (9.20%)	13 (13.00%)	
**NAC regimens**				
Anthracycline + taxane	443 (88.60%)	352 (88.00%)	91 (91.00%)	0.504
Others	57 (11.40%)	48 (12.00%)	9 (9.00%)	
**LA**				
Low	66 (13.20%)	51 (12.80%)	13 (13.00%)	1.000
High	434 (86.80%)	349 (86.20%)	87 (87.00%)	

Abbreviations: IQR, inter-quarter range; BMI, body mass index; IDC, invasive ductal carcinoma; ILC, invasive lobular carcinoma; pCR, pathologic complete response; HR, hormone receptors; BCS, breast conserving surgery; HER2, human epidermal growth factor receptor-2; NAC, neoadjuvant chemotherapy.

**Table 2:** NRI and IDI of the nomogram and AJCC TNM staging system in the DFS survival prediction for breast cancer patients received NAC.

Index	Training Cohort			Internal Validation Cohort			External Validation Cohort		
	Estimate	95% CI	*p*	Estimate	95% CI	*p*	Estimate	95% CI	*p*
**The nomogram vs. cTNM Staging**									
NRI									
1-year DFS	0.65	0.21–0.96		0.71	−0.43–1.55		1.33	0.62–1.57	
3-year DFS	0.55	0.22–0.81		0.45	−0.23–1.22		1.26	0.75–1.50	
5-year DFS	0.64	0.25–0.93		0.80	−0.20–1.40		0.83	0.33–1.24	
IDI									
1-year DFS	0.10	0.05–0.19	<0.001	0.05	−0.02–0.41	0.244	0.10	0.03–0.27	<0.001
3-year DFS	0.16	0.09–0.23	<0.001	0.04	−0.03–0.27	0.192	0.21	0.11–0.38	<0.001
5-year DFS	0.18	0.10–0.27	<0.001	0.11	−0.08–0.41	0.168	0.17	0.07–0.31	<0.001
**The nomogram vs. ypTNM Staging**									
NRI									
1-year DFS	0.60	0.28–0.90		0.51	−0.37–1.54		0.99	−0.05–1.37	
3-year DFS	0.60	0.18–0.84		0.17	−0.23–1.11		0.63	−0.16–1.26	
5-year DFS	0.68	0.07–0.88		0.14	−0.28–1.34		0.25	−0.19–0.91	
IDI									
1-year DFS	0.08	0.04–0.14	<0.001	0.05	−0.02–0.39	0.196	0.06	0.00–0.18	0.052
3-year DFS	0.10	0.05–0.17	<0.001	0.04	0.00–0.30	0.052	0.14	0.04–0.27	<0.001
5-year DFS	0.11	0.03–0.19	0.016	0.10	−0.06–0.37	0.128	0.12	0.04–0.23	<0.001

## Data Availability

The data presented in this study can be obtained upon request from the corresponding author. The data are not publicly accessible due to hospital copyright restrictions.

## Supplementary Materials

The following supporting information can be downloaded at: https://www.mdpi.com/article/10.3390/nu15194292/s1, Figure S1: Dot plot showing the standardized log-rank test statistic for DFS according to LA before NAC; Figure S2: Schoenfeld Residuals Plot for Cox Proportional Hazards Model of HR Status, LA, ypN Stage, and ypT Stage; Figure S3: The performance of risk stratification using the prognostic nomogram model in the internal validation cohort. (a) The distribution and median value of the risk scores. (b) The distribution of DFS, DFS status, and risk score. (c) The Kaplan–Meier curves for the DFS of patients which was divided into high-risk group and low-risk groups; Figure S4: The performance of risk stratification using the prognostic nomogram model in the external validation cohort. (a) The distribution and median value of the risk scores. (b) The distribution of DFS, DFS status, and risk score. (c) The Kaplan–Meier curves for the DFS of patients which was divided into high-risk group and low-risk groups; Table S1: The relationship between LA and clinicopathological characteristics in the training set; Table S2: Univariate and multivariate Cox regression analyses of DFS in the training cohort.
