# Supplementary material for: A Novel Albumin-Related Nutrition Biomarker Predicts Breast Cancer Prognosis in Neoadjuvant Chemotherapy: A Two-Center Cohort Study
Source: Nutrients. 2023 Oct 8;15(19):4292. doi: 10.3390/nu15194292 (PMC10574703; doi:10.3390/nu15194292)
Supplement: Supplementary file 1 [file nutrients-15-04292-s001.zip › nutrients-2617816-supplementary.pdf]

Supplementary Table S1 The relationship between LA and clinicopathological characteristics in the training set.

Characteristics	Total (n=400)	High-LA (n=349)	Low-LA (n=51)	*p*
**Age (years)**				
median (IQR)	48(40, 56)	48(40, 56)	49(45,55)	0.494
⩽50	236 (59.00%)	205 (58.70%)	31 (60.80%)	0.901
>50	164 (41.00%)	144 (41.30%)	20 (39.20%)	
**BMI(kg/m²)**				
median (IQR)	23.1(21.5, 25.0)	23.0(21.5, 25.0)	23.6(21.6, 25.5)	0.627
⩽25	302 (75.50%)	264 (75.60%)	38 (74.50%)	0.999
>25	98 (24.50%)	85 (24.40%)	13 (25.50%)	
**Menopausal status**				
Pre-menopausal	244 (61.00%)	210 (60.20%)	34 (66.70%)	0.463
Post-menopausal	156 (39.00%)	139 (39.80%)	17 (33.30%)	
**Histological types**				
IDC	372 (93.00%)	322 (92.30%)	50 (98.00%)	0.472
ILC	11 (2.80%)	11 (3.20%)	0 (0.00%)	
Others	16 (4.00%)	15 (4.30%)	1 (2.00%)	
Missing data	1 (0.20%)	1 (0.30%)	0 (0.00%)	
**Histological grade**				
I	5 (1.20%)	5 (1.40%)	0 (0.00%)	0.385
II	192 (48.00%)	172 (49.30%)	20 (39.20%)	
III	124 (31.00%)	104 (29.80%)	20 (39.20%)	
Missing data	79 (19.80%)	68 (19.50%)	11 (21.60%)	
**cT Stage**				
T1-T2	244 (61.00%)	215 (61.60%)	29 (56.90%)	0.621
T3-T4	156 (39.00%)	134 (38.40%)	22 (43.10%)	
**cN Stage**				
N0-N1	70 (17.50%)	66 (18.90%)	4 (7.80%)	0.081
N2-N3	330 (82.50%)	283 (81.10%)	47 (92.20%)	
**yT Stage**				
Tis/T0	75 (18.80%)	68 (19.50%)	7 (13.70%)	0.334
T1	107 (26.80%)	91 (26.10%)	16 (31.40%)	
T2	156 (39.00%)	140 (40.10%)	16 (31.40%)	
T3	37 (9.20%)	30 (8.60%)	7 (13.70%)	
T4	25 (6.20%)	20 (5.70%)	5 (9.80%)	
**yN Stage**				
N0	159 (39.80%)	138 (39.50%)	21 (41.20%)	0.925
N1	108 (27.00%)	93 (26.60%)	15 (29.40%)	
N2	75 (18.80%)	67 (19.20%)	8 (15.70%)	
N3	58 (14.50%)	51 (14.60%)	7 (13.70%)	
**pCR**				
No	335 (83.80%)	291 (83.40%)	44 (86.30%)	0.749

Characteristics	Total (n=400)	High-LA (n=349)	Low-LA (n=51)	*p*
Yes	65 (16.20%)	58 (16.60%)	7 (13.70%)	
**HR status**				
Negative	135 (33.80%)	114 (32.70%)	21 (41.20%)	0.125
Positive	263 (65.80%)	234 (67.00%)	29 (56.90%)	
Missing data	2 (0.50%)	1 (0.30%)	1 (2.00%)	
**HER2 status**				
Negative	220 (55.00%)	185 (53.00%)	35 (68.60%)	0.101
Positive	173 (43.20%)	158 (45.30%)	15 (29.40%)	
Missing data	7 (1.80%)	6 (1.70%)	1 (2.00%)	
**Ki-67(%)**				
median (IQR)	30(20, 53)	30(20,50)	40(20, 60)	0.505
≤ 14	52 (13.00%)	44 (12.60%)	8 (15.70%)	0.081
>14	340 (85.00%)	300 (86.00%)	40 (78.40%)	
missing data	8 (2.00%)	5 (1.40%)	3 (5.90%)	
**Lymphovascular invasion**				
No	238 (59.50%)	208 (59.60%)	30 (58.80%)	0.446
Yes	155 (38.80%)	136 (39.00%)	19 (37.30%)	
missing data	7 (1.80%)	5 (1.40%)	2 (3.90%)	
**Type of primary surgery**				
Mastectomy	363 (90.80%)	314 (90.00%)	49 (96.10%)	0.251
BCS	37 (9.20%)	35 (10.00%)	2 (3.90%)	

Abbreviations: IQR, inter-quarter range; BMI, body mass index; IDC, invasive ductal carcinoma; ILC, invasive lobular carcinoma; pCR, pathologic complete response; HR, hormone receptors; BCS, breast conserving surgery; HER2, human epidermal growth factor receptor-2; NAC, neoadjuvant chemotherapy

Supplementary Table S2 Univariate and multivariate Cox regression analyses of DFS in the training cohort.

Characteristic	Univariate analysis		Multivariate analysis	
	Hazard ratio(95%CI)	*p*	Hazard ratio(95%CI)	*p*
**Age (years)**				
≤50	1			
>50	0.76(0.68-1.33)	0.756		
**Menopausal status**				
Pre-menopausal	1			
Post-menopausal	0.89(0.63-1.25)	0.493		
**BMI(kg/m²)**				
≤25	1			
>25	1.02(0.69-1.49)	0.930		
**Histological types**				
IDC	1			
Others	0.95(0.52-1.76)	0.953		
**Histological grade**				
I	1			
II	0.61(0.08-4.38)	0.619		
III	0.89(0.12-6.47)	0.909		
**cT Stage**				
T1-T2	1			
T3-T4	1.51(1.09-2.11)	0.014		
**cN Stage**				
N0-N1	1			
N2-N3	1.03(0.67-1.60)	0.889		
**ypT Stage**				
Tis/T0	1		1	
T1-T2	2.23(1.19-4.16)	0.012	1.88(0.98-3.62)	0.058
T3-T4	3.57(1.81- 7.03)	<0.001	2.40(1.16-4.97)	0.019
**ypN Stage**				
N0	1		1	
N1	1.47(0.93-2.33)	0.097	1.51(0.93-2.47)	0.098
N2	2.26(1.42-3.60)	0.001	2.37(1.43-3.92)	<0.001
N3	2.94(1.82-4.74)	<0.001	3.24(1.92-5.47)	<0.001
**pCR**				
No	1			
Yes	0.41(0.21-0.80)	0.009		
**HR status**				
Negative	1		1	
Positive	0.64(0.46-0.90)	0.011	0.52(0.36-0.74)	<0.001
**HER2 status**				
Negative	1			
Positive	1.09(0.78-1.54)	0.606		

Characteristic	Univariate analysis		Multivariate analysis	
	Hazard ratio(95%CI)	*p*	Hazard ratio(95%CI)	*p*
**Ki-67(%)**				
≤ 14	1			
>14	1.70(0.99-2.92)	0.053		
**Lymphovascular invasion**				
No	1			
Yes	1.40(0.99-1.96)	0.055		
**LA**				
High	1		1	
Low	1.99(1.30-3.06)	0.002	2.00(1.29-3.11)	0.002

Abbreviations: IQR, inter-quarter range; BMI, body mass index; IDC, invasive ductal carcinoma; pCR, pathologic complete response; HR, hormone receptors; HER2, human epidermal growth factor receptor-2

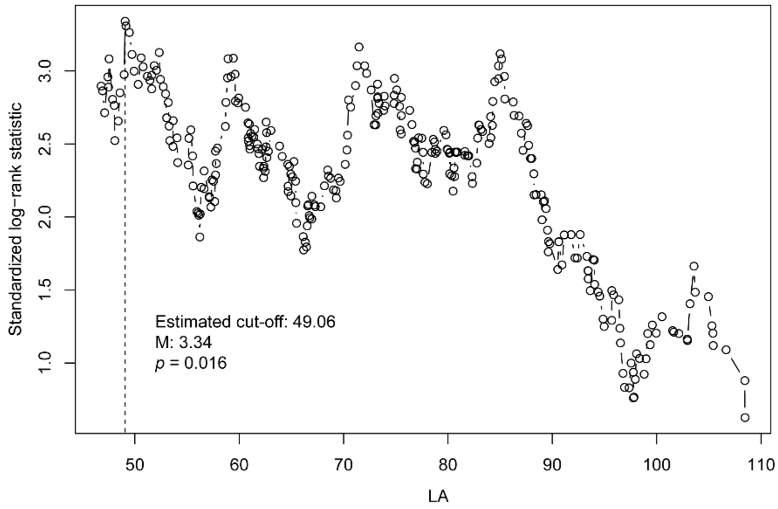

Supplementary Figure S1. Dot plot showing the standardized log-rank test statistic for DFS according to LA before NAC. Abbreviations: DFS: disease-free survival; M: standardized log-rank test statistic

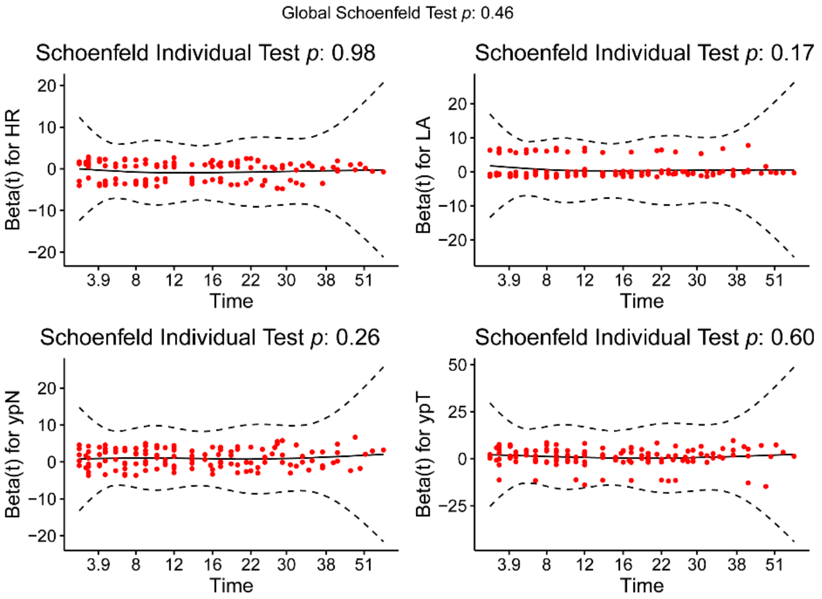

Supplementary Figure S2. Schoenfeld Residuals Plot for Cox Proportional Hazards Model of HR Status, LA, ypN Stage and ypT Stage. Abbreviations: HR, hormone receptors

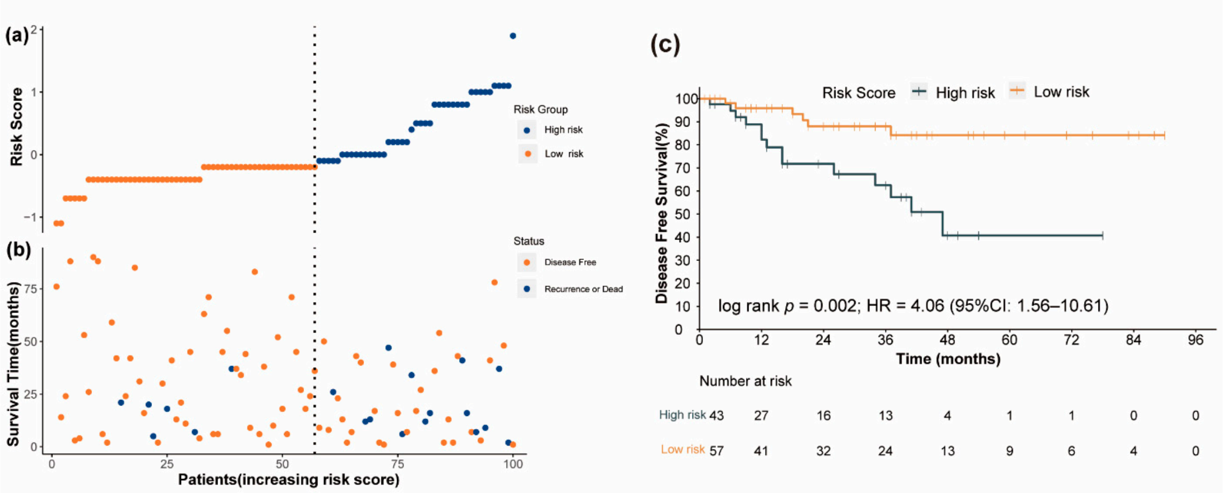

Supplementary Figure S3. The performance of risk stratification using the prognostic nomogram model in the internal validation cohort ("Number at risk" represents the remaining number of individuals exposed to the outcome risk at the corresponding time point). (a) The distribution and median value of the risk scores. (b) The distribution of DFS, DFS status, and risk score. (c) The Kaplan–Meier curves for the DFS of patients who were divided into high-risk and low-risk group.

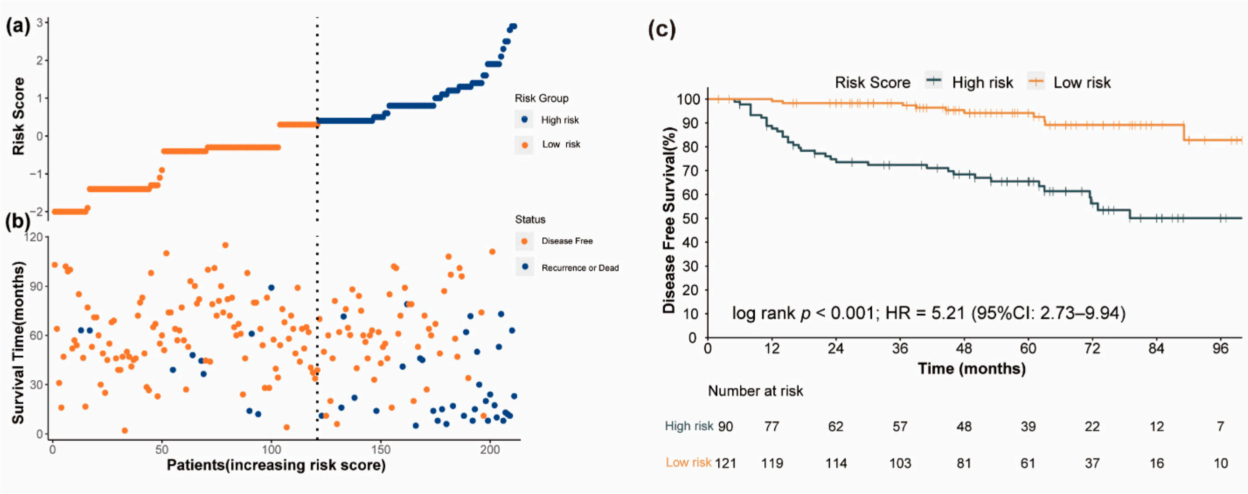

Supplementary Figure S4. The performance of risk stratification using the prognostic nomogram model in the external validation cohort ("Number at risk" represents the remaining number of individuals exposed to the outcome risk at the corresponding time point). (a) The distribution and median value of the risk scores. (b) The distribution of DFS, DFS status, and risk score. (c) The Kaplan–Meier curves for the DFS of patients who were divided into high-risk and low-risk group.
